# Dysregulated MicroRNA Fingerprints and Methylation Patterns in Hepatocellular Carcinoma, Cancer Stem Cells, and Mesenchymal Stem Cells

**DOI:** 10.3389/fcell.2019.00229

**Published:** 2019-10-17

**Authors:** Mohamed A. Nasr, Radwa Ayman Salah, M. Abd Elkodous, Shimaa E. Elshenawy, Nagwa El-Badri

**Affiliations:** Center of Excellence for Stem Cells and Regenerative Medicine (CESC), Zewail City of Science and Technology, 6th of October City, Egypt

**Keywords:** miRNA, cancer stem cell, methylation, mesencymal stem cells, hepatocellular carcinoma

## Abstract

Hepatocellular carcinoma (HCC) is one of the top causes of cancer mortality worldwide. Although HCC has been researched extensively, there is still a need for novel and effective therapeutic interventions. There is substantial evidence that initiation of carcinogenesis in liver cirrhosis, a leading cause of HCC, is mediated by cancer stem cells (CSCs). CSCs were also shown to be responsible for relapse and chemoresistance in several cancers, including HCC. MicroRNAs (miRNAs) constitute important epigenetic markers that regulate carcinogenesis by acting post-transcriptionally on mRNAs, contributing to the progression of HCC. We have previously shown that co-culture of cancer cells with mesenchymal stem cells (MSCs) could induce the reprogramming of MSCs into CSC-like cells. In this review, we evaluate the available data concerning the epigenetic regulation of miRNAs through methylation and the possible role of this regulation in stem cell and somatic reprogramming in HCC.

## Hepatocellular Carcinoma

Hepatocellular carcinoma (HCC) is the most frequent primary malignancy of the liver. It is the third leading cause of mortality associated with cancer worldwide (Yang and Roberts, 2010; Dhanasekaran et al., 2012). HCC is a multifactorial disease that is influenced by several risk factors. It typically develops as a result of underlying liver disease and is commonly associated with cirrhosis (Huang et al., 2013). The major HCC-risk factors include viral infection with hepatitis B virus (HBV) and hepatitis C virus (HCV), which leads to liver cirrhosis and accounts for 75% of HCC cases (El-Serag, 2002). Other factors attributed to HCC include alcohol abuse, intake of food contaminated with aflatoxin and toxic chemical exposure, including dimethylformamide, dimethylacetamide, trichloroethylene, tetrachloroethylene, carbon tetrachloride and chloroform (Malaguarnera et al., 2012). In addition, Obesity is one of the highly recent factors that plays a significant role in developing non-alcoholic fatty liver disease (NAFLD) (Cholankeril et al., 2017). It can progress in many stages starting with lipid deposition in hepatocytes' cytoplasm and can lead to non-alcoholic steatohepatitis (NASH) (Marrero et al., 2002; Guzman et al., 2008; Reddy et al., 2012; White et al., 2012). NASH is the severe stage of NAFLD indicated by hepatocyte injury, uncontrolled inflammation, hepatocyte ballooning, cell death, infiltration of inflammatory cells, and collagen deposition (Guzman et al., 2008; Reddy et al., 2012). NASH has been determined to be one of the important events in promoting hepatic carcinogenesis (Ip and Wang, 2014).

Tissue damage and fibrosis result from chronic inflammation and oxidative stress, leading to cirrhosis and eventually tumor initiation, progression, and even metastasis (Lau and Lai, 2008; Shariff et al., 2009; Cabrera and Nelson, 2010). Although only ~10–20% of HCC patients are eligible for surgical interference at the time of diagnosis, liver transplantation remains the first choice for treatment (Ji et al., 2009a). Furthermore, patients suffer a high frequency of relapse, and in patients who experience curative resection, the 5-year survival rate is 30–40% (Budhu et al., 2008). The low detection and high recurrence rates for the curable stages of HCC have increased interest in investigations of the molecular mechanisms underlying this disease (He et al., 2015).

## Cancer Stem Cells (CSCs)

The failure of conventional treatments to completely eliminate invasive tumor cells is thought to be due to the presence of a small subset of cancer cells, termed CSCs, which are accountable for cancer progression, metastasis, recurrence, and drug resistance. CSCs have been classified as immortal tumor-initiating cells which have pluripotent and self-renewal capacity (Chen et al., 2013). CSCs have been identified in numerous solid tumors, such as breast cancer, colon cancer, and HCC (Szotek et al., 2006; O'Brien et al., 2007; Kawai et al., 2015). CSCs were found to have a main contribution in tumor heterogeneity and to contribute to drug resistance (Beck and Blanpain, 2013; Bedard et al., 2013; Klein, 2013; Meacham and Morrison, 2013). While the origin of CSCs remains unclear, the proposed mechanisms for their generation include cell fusion, genetic mutations in stem cells, and regulatory factors within the tumor microenvironment (TME) (Bu and Cao, 2012). In addition, signaling pathways and genes that regulate stem cell differentiation, as Wnt/β-catenin, transforming growth factor β (TGF-β), and microRNAs (miRNAs), could contribute to the control and maintenance of CSC differentiation (Bedard et al., 2013; Meacham and Morrison, 2013). The Wnt/β-catenin signaling pathway seems to play major roles in the development of CSCs and in self-renewal, tumorigenesis, and cancer chemoresistance (Espada et al., 2009; Eaves and Humphries, 2010; Mohammed et al., 2016).

## Characteristics of miRNAs

MiRNAs are small non-coding RNA molecules consisting of 21−23 nucleotides. They control gene expression by base pairing with the messenger RNAs (mRNAs) (Lu et al., 2005; Griffiths-Jones et al., 2006). Transcripts are regulated through either degradation or translational repression (Bartel, 2004). Full complementarity between a miRNA and an mRNA results in full degradation of the target mRNA. However, defects in perfect complementarity leads to less translation of the target gene without affecting the level of mRNA (Lewis et al., 2005; Cummins and Velculescu, 2006). MiRNAs target up to 90% of human genes (Miranda et al., 2006) and can be found in exons or introns of coding or non-coding genes, with their transcription dependent on genomic localization (Baskerville and Bartel, 2005; Lin et al., 2006). Although miRNAs have their own promoters and are self-sufficiently expressed some miRNAs that share the same transcriptional regulation are ordered in clusters. Hundreds of miRNAs have been known by molecular cloning and bioinformatics approaches in plants and animals (Bushati and Cohen, 2007; Liu et al., 2014). Interestingly, a subgroup of miRNAs, namely, epi-miRNAs, control the expression of epigenetic marks, such as DNA methyltransferases (DNMTs), histone deacetylases (HDACs), and polycomb genes, either directly or indirectly. DNA methylation has a key role in gene expression regulation via maintaining the stability of gene silencing. In mammals, DNA methylation takes place by covalent modification of cytosine residues through the addition of a methyl group to the fifth position of a cytosine ring, particularly in the CpG dinucleotides. This process is mediated by members of the DNMTs enzymes family (Chuang and Jones, 2007). Therefore, miRNAs function as both genetic and epigenetic regulators (Valeri et al., 2009).

miRNAs control many cellular processes in eukaryotes, such as rate of growth, development, differentiation potential, cell cycle progression, and apoptosis, and their abnormal expression affects many human diseases (Valeri et al., 2009; Krol et al., 2010; Wahid et al., 2010; Pritchard et al., 2012). In addition to serving as essential players in tumor development, miRNAs have a role as possible biomarkers for cancer (Calin and Croce, 2006). Indeed, miRNA profiles reflect the stages of tumors and their developmental lineages (George and Mittal, 2010). MiRNAs have been found to modulate CSCs and metastasis. They can also act as oncogenes and tumor suppressors. Due to their functions as oncogenes and tumor suppressor genes, these miRNAs have been referred to as oncomirs (George and Mittal, 2010).

## miRNAs and HCC

Recent studies on liver miRNAs investigated the overexpression of specific miRNAs or the inhibition of other miRNAs both *in vitro* and *in vivo*. These studies showed the crucial biological roles of miRNAs for proper liver function (Takata et al., 2013). In HCC, Murakami et al. initially reported dysregulated miRNA expression, with four miRNAs, namely, miR-18, miR-92, miR-20, and precursor miR-18 being inversely associated with the extent of HCC development (Murakami et al., 2006). Later, several studies confirmed that miRNAs play an essential regulatory role in hepatic carcinogenesis progression and malignant transformation. Some miRNAs showed abnormal expression during the progression of liver cancer (Zhao et al., 2009). The expression of some miRNAs was shown to influence HCC development via dysregulation of a number of cancer-associated molecular pathways, including TGF-β, *p53, WNT/*β*-catenin, P13K/AKT/mTOR, RAS/MAPS, MET, and MYC* (Negrini et al., 2011). Many oncogenic miRNAs have shown aberrant expression in HCC, including miR-1275 (Shaalan et al., 2018), miR-17-5p (Habashy et al., 2016), miR-96-5p, miR-182-5p (Assal et al., 2015), miR-155 (El Tayebi et al., 2015), and miR-181a (Lashine et al., 2011). Other tumor suppressor miRNAs involved in HCC include miR-34a (Yacoub et al., 2016), miR-486-5p (Youness et al., 2016), miR-615-5p (El Tayebi et al., 2012), and miR-Let7i (Fawzy et al., 2016). Genome-wide approaches have identified hundreds of miRNAs in HCC tumor tissues that were to be dysregulated compared to non-tumor tissues (Borel et al., 2012). MiR-122 is among many unique and well-studied dysregulated miRNAs that are highly expressed specifically in human liver. In HCC patients, a shorter recurrence time were attributed to lower levels of miR-122. While elevated expression of cyclin G1, a target of miR-122, was associated with a lower survival rate. Moreover, miR-122 acts as a tumor suppressor in HCC, and was subsequently reported to be downregulated in around 70% of cases (Callegari et al., 2013). MiR-221 is another critical oncogenic miRNA that is upregulated in 70–80% of HCC cases. Its overexpression leads to enhanced proliferation potential, migration, invasion, rate of growth, and decreased the rate of apoptosis in HCC patients (Fornari et al., 2008). Additionally, miR-221 modulates several gene targets involved in cancer-related pathways, includin*g PTEN* (*P13K/AKT/mTOR*), *CDKN1B/p27*, and *CDKN1C/p57* (Fornari et al., 2008; Garofalo et al., 2009).

Due to their non-invasive detection, good specificity, and sensitivity, miRNAs are considered effective biomarkers for HCC (Shen et al., 2016). MiR-155-5p, miR-206, miR-21-5p, and miR-212-3p. MiR-155-5p and miR-21-5p which are reported as biomarkers for the prognosis of HCC in tissues, were found to have upregulated expression levels. On the other hand others were down-regulated (Han et al., 2013; Wang et al., 2014; Yunqiao et al., 2014; Tu et al., 2015). Circulating miR-122-5p and miR-16-5p could be used as presumed biomarkers for HCC. MiR-122-5p and miR-16-5p belong to this group which were particularly detected to be up and down-regulated, respectively (Cho et al., 2015; El-Abd et al., 2015).

Most often, elevated expression of miR-18b-5p, miR-200a-3p, miR-200b-3p, miR-21-5p, miR-224-5p, and miR-29-5p in tissue were mostly reported to be HCC. In addition, miR-139-5p was down-regulated. Therefore, they were beneficial for diagnosis of HCC (Zhu et al., 2012; Murakami et al., 2013; Dhayat et al., 2014; Han et al., 2014; Li T. et al., 2014; Amr et al., 2016).

Circulating miRNAs were proposed as prognostic biomarkers and reported to be linked to tissue invasion and metastasis. Those biomarkers included miR-122-5p, miR-17-5p, miR-182-5p, miR-21-5p, miR-24-3p, and miR-331-3p, all were up-regulated in the group reported to have a low-survival rate (Zheng et al., 2013; Meng et al., 2014; Chen et al., 2015; Wang L.-J. et al., 2015; Xu Y. et al., 2015). Meanwhile, the serum miR-150-5p was highly expressed in HCC patients after surgical operation, however following tumor relapse its expression levels were reversed (Yu F. et al., 2015).

In tissues, high expression of miR-150-5p and miR-29a-5p in combination of low expression of miR-101-3p, miR-126-3p, miR-127-3p, miR-139-5p, and miR-214-3p have tumor-suppressor roles and consequently have potential use as diagnostic biomarkers for HCC (Zhu et al., 2012; Han et al., 2014; Li T. et al., 2014; Peveling-Oberhag et al., 2014; Xie et al., 2014; Zhou et al., 2014; Wang S. et al., 2015). The association between the circulating miR-101-3p, miR-122-5p, miR-125b-5p, miR-139-5p, miR-150-5p, miR-16-5p, miR-181a-5p, miR-199a-3p, miR-199a-5p, miR-203a-3p, miR-21-5p, miR-22-3p, miR-29b-3p, miR-375, let-7b-5p, and tumor suppressor render them potential biomarkers for differentiating HCC from healthy controls (Zhou J. et al., 2011; Luo et al., 2013; Li T. et al., 2014; Tan et al., 2014; Xie et al., 2014; Chen et al., 2015; Jiang et al., 2015; Wang S. et al., 2015; Yin et al., 2015; Yu F. et al., 2015; Hung et al., 2016). Contrarily, miR-101-3p, miR-122-5p, miR-125b-5p, miR-130a-3p, miR-146a-5p, miR-214-3p, and miR-99a-5p were known as tumor suppressors in HCC and played the role of prognostic indicators for HCC (Zhang et al., 2012; Wang et al., 2013; Li B. et al., 2014; Rong et al., 2014; Tsang et al., 2014; Xie et al., 2014; Xu Q. et al., 2015). Serum miR-1-3p, miR-101-3p, miR-122-5p, miR-150-5p, miR-203a-3p, and miR-30c-5p were linked to tumor suppression, and new independent parameters of overall survival in HCC (Köberle et al., 2013; Xie et al., 2014; Cho et al., 2015; Liu D. et al., 2015; Xu Y. et al., 2015; Yu F. et al., 2015).

As miRNAs expression levels can be used as biomarkers for HCC diagnosis and prognosis, miRNA specific methylation patterns are of importance for therapeutic applications as well. Acting as a tumor suppressor miRNA, decreased expression of miR-10a due to hypermethylation can be used as a biomarker for early HCC diagnosis and risk assessment (Shen et al., 2012). Furthermore, some miRNAs methylation patterns can be HCC cell-specific and therefore used as diagnostic biomarkers. Such miRNAs cell-specific diagnostic methylation patterns include the hypermethylation of miR-129-2, miR-34a, and miR-148a (Anwar et al., 2013; Lu et al., 2013). Also, hypermethylation of mir-9-1 has been shown to be a biomarker for poor diagnosis and aggressiveness (Anwar et al., 2013). In addition to their implications in diagnosis and prognosis, miRNA specific aberrant methylation patterns can be used for therapeutic applications. For example, administration of miR-124, which is hypermethylated in HCC, stopped HCC progression in animal models and was considered safe. Moreover, sorafenib (anti-cancer drug) increased the expression of miR-1274, which is hypermethylated in HCC, leading to an increased response to therapy (Zhou C. et al., 2011).

## miRNAs and CSCs in HCC

miRNAs play essential roles in regulating CSCs (Garg, 2015), and in regulating apoptosis in CSCs by acting on mRNAs of apoptosis proteins or regulating mRNAs that are downstream targets in specific apoptotic pathways. These control mechanisms aid in the regulation of metastasis, drug resistance, tumor invasion, pluripotency, and self-renewal potential.

The tumorigenicity of liver CSCs was found to be significantly suppressed by inhibition of miR-181. This miRNA regulates the differentiation potential of liver CSCs through activating transcription factors, including caudal homeobox gene 2 (CDX2) and GATA6, and negatively regulating the Wnt/β-catenin pathway via nemo-like kinase (NLK) (Ji et al., 2009b; Leal and Lleonart, 2013; Bessède et al., 2014). MiR-Let-7 and miR-Lin28 have been reported to be related to the rate of growth and metastasis of HCC. Lin28 is highly expressed in normal embryonic stem cells (ESCs). It maintains the self-renewal of liver CSCs by inhibiting the interaction of Let-7 with the mature miRNA. Let-7 degradation, which is caused by excessively active Lin28 and c-MYC, dis-equilibrates liver CSCs, leading to accelerated growth and metastasis of HCC (Heo et al., 2008). MiRNAs positively regulate liver CSCs via high expression of EpCAM, which is a prominent marker of liver CSCs. This upregulation is mediated by inhibition of TGF-β by downstream transcription factors of miR-18, such as CDX2, GATA6, and NLK. The EpCAM intracellular domain (EpICD) enters the nucleus and induces overexpression of cyclin D1, c-MYC, and miR-181 after binding to LIM domain protein 2 (FHL2), β-catenin and lymphoid enhancer factor 1 (Lef-1) (Ji et al., 2009b). Another group showed that TGF-β1 downregulate TP53INP1 by targeting miR-155 and promote epithelial-mesenchymal transition (EMT) and liver CSC phenotypes (Liu F. et al., 2015). The Wnt/β-catenin signaling pathway that regulates tumor heterogeneity is mainly related to miRNA, but the mechanism by which this balance between liver CSCs and cancer cells is maintained has not been elucidated.

Based on previous studies, some miRNAs expression was reported to be dysregulated in both HCC and CSCs. In Table 1, we compiled the mutually dysregulated miRNAs to establish the links between these miRNAs and the initiation and progression of HCC.

**Table 1:** miRNAs whose expression was reported to be dysregulated in HCC and CSCs.

**miRNA**	**Expression and biological function in HCC**	**Expression and biological function in CSCs**
miR-Let-7a	Downregulated (Connolly et al., 2008) MiR-Let-7 family act as tumor suppressors via regulating expression of oncogenes including RAS, COL1A2, and AT-hook 2 high mobility group (Johnson et al., 2005; Shi et al., 2017)	Downregulated (Yata et al., 2015) The let-7 miRNA family has been reported to maintain the state of differentiation and self-renewal inhibition of CSCs by targeting Lin28, H-RAS, and HMGA2 (Ali Hosseini Rad et al., 2013; Sun et al., 2016)
miR-Let-7b miR-Let-7c miR-Let-7d miR-Let-7e miR-Let-7f miR-Let-7g	Downregulated (Gramantieri et al., 2007) Act as tumor suppressors by targeting mRNA of RAS, COL1A2, and AT-hook 2 group	Downregulated (Peter, 2009) miR-Let-7 family is involved in the differentiation of CSCs and maintenance of stemness by targeting Lin28 which have a negative effect on Let-7, and inhibits the expression of H-RAS and HMGA2 (Ali Hosseini Rad et al., 2013)
miR-9	Upregulated (Wang et al., 2008) MiR-9 increases growth and metastasis (Chen et al., 2015). It targets directly the 3′UTR of PPAR alpha and regulates its expression levels in liver cancer cells. MiR-9 is highly over expressed in liver cancer patients. It acts as tumor suppressor, and could have a therapeutic effect (Chen et al., 2015)	Upregulated (Schraivogel et al., 2011) miR-9 is responsible for maintaining stemness and promotion of CD 133^+^ cell proliferation by targeting CAMTA 1 (Takahashi et al., 2014; Wang L.-J. et al., 2015)
miR-16	Upregulated (Huang et al., 2009) MiR-16 targets and regulates Bcl-2 and suppresses Bcl-2 protein expression by binding to the 3′UTR of Bcl-2 mRNA. Bcl-2 is one of the well-known anti apoptotic protein members in Bcl-2 family that control cancer cells response to drugs (Wang et al., 2018). RHepG2 cells, the multidrug resistant subgroup of human HCC HepG2 cells were reported to have higher levels of miR-16 and P-glycoprotein which is associated with multidrug resistance and lower level of Bcl-2 (Fregni et al., 2018)	Upregulated (Caruso et al., 2012) miR-16 regulates proliferation and self-renewal by targeting bmi1 (B lymphoma Mo-MLV insertion region 1 homolog) (Shimono et al., 2015)
miR-17	Upregulated (Huang et al., 2009) The miR-17-92 cluster is the first oncogenic miRNAs identified in human (El-Badawy et al., 2017). This cluster targets thrombospondin-1 (TSP1), proangiogenic targets, and connective tissue growth factor (CTGF) to increase the potential of tumor angiogenesis (Sung et al., 2013)	Upregulated (Schraivogel et al., 2011) Promotion of CD 133^+^ cell proliferation by targeting CAMTA 1 (Takahashi et al., 2014)
miR-20a	Upregulated (Connolly et al., 2008) Involved in proliferation and recurrence of HCC (Zheng et al., 2013). MiR-20 a reduced the endogenous level of myeloid cell leukemia sequence 3′UTR Mcl-1 protein in HCC (Liu et al., 2012)	Upregulated (Caruso et al., 2012) Regulating and enhancing stemness properties (Yu F. et al., 2015)
miR-21	Upregulated (Connolly et al., 2008) Involved in progression and metastasis (Xie et al., 2014). miR-21 inhibited KLF5 gene by targeting its 3′-UTR. KLF5 gene play a role in cancer as a tumor inhibitor (Wang et al., 2012)	Upregulated (Caruso et al., 2012) enhance stemness properties of CSCs by targeting TGFβR2 (Ali Hosseini Rad et al., 2013)
miR-24	Upregulated (Huang et al., 2009) Has a role in metastasis and invasion (Zhou et al., 2014) miRNA-24 binds to the 3′-UTR of p53 mRNA and down regulates its expression	Upregulated (Roscigno et al., 2017) Involved in maintaining stemness markers It has been postulated that miR-24 survived stem cells from apoptosis in the hypoxia conditions through an FIH1–HIFα pathway as it have HIF binding site (Peveling-Oberhag et al., 2014)
miR-27a	Upregulated (Connolly et al., 2008) MiRNA-2a regulates PPAR-γ expression through promoting HCC cell proliferation	Upregulated (Caruso et al., 2012) Promotes angiogenesis and metastasis through targeting ENPP1 (Shimono et al., 2015)
miR-29c	Downregulated (Su et al., 2009) miR-29c targets SIRT1 oncogene thus acting as a tumor suppressor (Hung et al., 2016)	Upregulated (Caruso et al., 2012)
miR-96	Upregulated (Wang et al., 2008) MiR-96 over expression targets SOX6 that regulates proliferation potential, invasion and migration (Liu Y. et al., 2011)	Downregulated (Shimono et al., 2015)
miR-34a	Downregulated (Sun et al., 2017; Xia et al., 2017; Ren et al., 2018) Involved in inhibition of invasion and migration potential Through c-Met signaling pathway (Rong et al., 2014) miR-34a targeted c-Met resulting in decreasing the mRNA and protein levels; thus, decrease phosphorylation of extracellular signal-regulated kinases 1 and 2 (Li N. et al., 2009)	Downregulated (Wheeler et al., 2009; Stadler et al., 2010) Involved in inhibiting the self-renewal and metastasis of CSCs (Tsang et al., 2014) and suppression of asymmetric cell division in CSCs by targeting NOTCH 1 (Wang et al., 2013; Takahashi et al., 2014)
miR-93	Upregulated (Thurnherr et al., 2016) MiR-93 over expression *in-vitro* enhanced HCC cell migration and invasion by targeting Programmed cell death 4 (PDCD4) gene (Zhang et al., 2012)	Downregulated (Caruso et al., 2012)
miR-495	Upregulated (Yang et al., 2013) miR-495 targets IGFIR and regulates ERK and AKT pathways, therefore inhibiting invasion and proliferation potential of HCC cells (Kim et al., 2012)	Upregulated (Hwang-Verslues et al., 2011) Enhance stemness properties of CSCs by targeting REDD1 (Ali Hosseini Rad et al., 2013)
miR-1246	Downregulated (El-Halawany et al., 2015) miR-1246 enhances the cell migration by targeting 3′UTR of the cell adhesion molecule 1 and downregulating its expression (Ma et al., 2010)	Upregulated (Eshelman and Yochum, 2016; Zhang et al., 2016) Maintains properties of CSCs by targeting Wnt/beta-catenin pathway (Lou et al., 2018)
miR-210	Upregulated (Yang et al., 2016) miR-210 targets SMAD4 and STAT6 to promote tumor angiogenesis (Huang et al., 2008)	Upregulated (Bao et al., 2012) Promotes metastasis, proliferation, and self-renewal of CSCs by targeting E-cadherin (Tang et al., 2018)
miR-18	Upregulated (Liu et al., 2017) The inhibition of miR-18 enhances the migration of HCC by targeting Smad2 (Li L. et al., 2015)	Upregulated (Turchi et al., 2013) Modulates tumorigenesis (Mens and Ghanbari, 2018)
miR-191	Upregulated (He et al., 2011) miR-191 promotes proliferation, tumor growth of HCC cells, and apoptosis by targeting TIMP3, TMC7, SOX4, and IL1A (Chang et al., 2012)	Upregulated (Xu W. et al., 2015)
miR-7	Downregulated (Yu et al., 2016) miR-7 targets oncogenes such as Cdr 1 and acts tumor suppressor (Lu et al., 2013)	Downregulated (Zhang et al., 2014) Inhibits metastasis by targeting SETDB1 (Shimono et al., 2015)
miR-150	Downregulated (Thurnherr et al., 2016) miR-150 acts as tumor suppressor by inhibiting GAB1 expression and downregulating ERK activation	Upregulated (Liu D. Z. et al., 2015) increases proliferation by targeting Wnt signaling pathway (Shimono et al., 2015)
miR-145	Downregulated (Thurnherr et al., 2016) Downregulated (Thurnherr et al., 2016) miR-145 regulates growth rate and proliferation and the re-expression of miR-145 resulting in cell apoptosis. It has been reported that insulin-like growth factor (IGF) is an important oncogenic pathway in HCC. Meanwhile, miR-145 targets insulin receptor substrate (IRS1)-1, IRS2, and insulin-like growth factor 1 receptor (Law et al., 2012)	Downregulated (Yu Y. et al., 2015; Zhou et al., 2017) Targets ROCK1, Oct-4, Sox2, and Klf 4 genes and inhibits tumorigenesis, invasion, and stemness (Shi et al., 2017)
miR-101	Downregulated (Su et al., 2009) Involved in promoting apoptosis and suppressing tumorigenicity (Jiang et al., 2015) via targets RAB5A, STMN1, and ATG4D (Xu Y. et al., 2013)	Upregulated (Caruso et al., 2012)
miR-141	Downregulated (Gramantieri et al., 2007) MiR-141 targets Tiam1 genes and inhibits HCC cells migration, proliferation and invasion potential *in-vitro* (Schoolmeesters et al., 2009)	Downregulated (Gregory et al., 2008) inhibits the proliferation of CSCs by suppressing Wnt signaling pathway (Shimono et al., 2015)
miR-142	Downregulated (Gramantieri et al., 2007)	Upregulated (Caruso et al., 2012; Chapnik et al., 2014) Inhibits the proliferation of CSCs by suppressing Wnt signaling pathway (Shimono et al., 2015)
miR-155	Upregulated (Wang et al., 2008) Enhances proliferation, growth, and tumorigenesis and decreases apoptosis (Gramantieri et al., 2007; Peter, 2009)	Upregulated (Caruso et al., 2012)
miR-183	Upregulated (Wang et al., 2008) Regulates carcinogenesis (Takahashi et al., 2014). MiR-183 suppress apoptosis in HCC cells through targeting Programmed cell death 4 (PDCD4). PDCD4 mediates its proapototic function in human HCC by being involved in the TGF-β1-induced apoptotic pathway (Li et al., 2010)	Downregulated (Dambal et al., 2015; Leung et al., 2015) Maintains EMT and self-renewal of CSCs by targeting SNAI2, SMAD4, and bmi1 (Shimono et al., 2015)
miR-185	Downregulated (Huang et al., 2009) In HCC: miR-185 induces HCC proliferation by targeting the DNTM1 3′UTR luciferase (Chang et al., 2014)	Upregulated (Caruso et al., 2012)
miR-194	Downregulated (Huang et al., 2009) miR-194 induces apoptosis by targeting MAP4K4 (Peuget et al., 2014)	Upregulated (Caruso et al., 2012) maintains self-renewal by targeting bmi1 (Shimono et al., 2015)
miR-200a	Downregulated (Murakami et al., 2006) Involved in enhancing proliferation and carcinogenesis (Caruso et al., 2012). Meanwhile, it regulates the invasion and migration of HCC cells by targeting Foxa2 (Chen et al., 2017)	Downregulated (Pode-Shakked et al., 2013) Involved in inhibiting EMT, BMI1, Sox2, Klf4, and Notch signaling, and reducing the stemness properties and mammosphere formation of CSCs by targeting ZEB1 and ZEB 2, SIP1, BMI-1, and Klf4 (Ali Hosseini Rad et al., 2013; Shimono et al., 2015)
miR-200b	Downregulated (Huang et al., 2009) Involved in enhancing proliferation and carcinogenesis (Caruso et al., 2012) miR-200b suppressed the expression of BMI1 and ZEB1, moreover ZEB1 promotes CD13, CD24, and EPCAM resulting in the upregulation of CD13 and CD24 so, the miR-200-ZEB1 circuit regulates stemness in HCC and differentiates between HCC contains CD13^+^/CD24^+^ CSCs from EpCAM + CSCs (Tsai et al., 2017)	Downregulated (Pode-Shakked et al., 2013) Involved in inhibiting EMT, BMI1, Sox2, Klf4, and Notch signaling, and reducing the stemness properties of CSCs by targeting ZEB1, SIP1, Bmi-1, and Klf4 (Ali Hosseini Rad et al., 2013; Shimono et al., 2015)
miR-214	Downregulated (Wang et al., 2008) MiR-214 acts as a suppressor to HCC by downregulating β-catenin signaling pathway (Nie et al., 2011)	Upregulated (Zhang et al., 2006) Maintains properties of CSCS by targeting CTNNB1 (Lou et al., 2018)
miR-215	Downregulated (Su et al., 2009) miR-215 showed significant upregulation in HCC serum, and thus functions as a biomarker for early diagnosis in HCC patients miR-215 is significantly correlated with the important genes in Wnt/β-catenin pathway including. β-catenin, APC, and c-myc (Ashmawy et al., 2017)	Upregulated (Caruso et al., 2012) Acts as tumor suppressor (Ullmann et al., 2019) Mechanistically, miR-215 targets by a cell cycle-regulated nuclear and centrosome protein, which suppress the miRNA resulted in the induction of P53, G2 arrest, and P21 and decrease the proliferation (Song et al., 2010)
miR-221	Upregulated (Huang et al., 2009) Involved in enhancing proliferation and tumorigenicity (Sun et al., 2017; Ren et al., 2018)	Upregulated (Shimono et al., 2009) Promotes progression of CSCs by targeting PTEN (Li et al., 2017)
miR-222	Upregulated (Huang et al., 2009) Involved in enhancing tumorigenesis (Ren et al., 2018) and increase the growth rate by targeting the cyclin-dependent kinase inhibitor p27 (Song et al., 2017)	Upregulated (Shimono et al., 2009) Promotes progression of CSCs by targeting PTEN (Li et al., 2017)
miR-424	Downregulated (Su et al., 2009) MiR-424 is involved in tumorigenesis of HCC by suppression of c-Myb. It directly targets the 3UTR of c-Myb and induces inhibition of proliferation and invasion in HCC cells (Zhang et al., 2011)	Downregulated (Yata et al., 2015)
miR-34a	Downregulated (Peveling-Oberhag et al., 2014; Xie et al., 2014; Yu F. et al., 2015) MiR-34a induces apoptosis of HCC and decreases its proliferation by targeting the expression of HDAC1. It acts as a 3′UTR to regulate its expression on HCC cells (Peveling-Oberhag et al., 2014)	Downregulated (Zhou et al., 2014; Wang S. et al., 2015) Inhibits self-renewal properties of CSCs by targeting CD44 and Notch1 (Ali Hosseini Rad et al., 2013)
miR-378	Downregulated (Xu Y. et al., 2015) Rs1076064 (pri-miR-378) variant genotypes contributed to the expression of the miR-378 in decreasing the risk of HCC (Mei et al., 2013)	No data
miR-24	Upregulated (Li T. et al., 2014) In HCC: miR-24 regulates oncogenes by binding to 3′-UTR of P53 (Hassan et al., 2010)	Upregulated (Wang L.-J. et al., 2015) Promotes apoptosis resistance through regulating BimL (Roscigno et al., 2017)
miR-29c	Downregulated (Xu Y. et al., 2015) In HCC: miR-29c targets oncogenic by binding to 3′UTR in SIRT1 (Sun et al., 2009)	Upregulated (Murakami et al., 2013)

**Figure 1:**
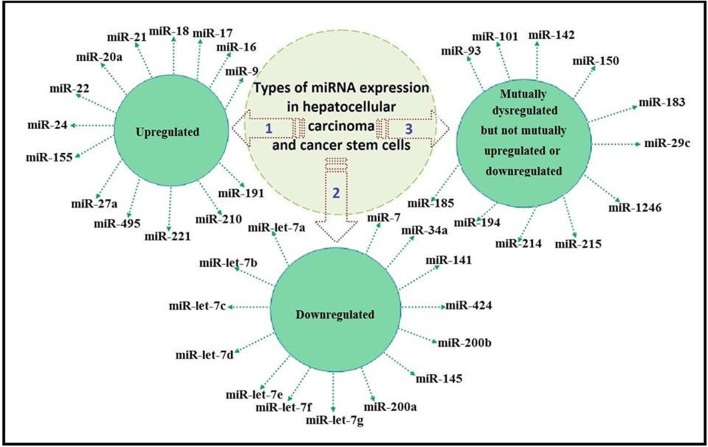
Classification of miRNA expression as mutually upregulated, mutually downregulated, and mutually dysregulated but not mutually upregulated or downregulated.

We classified the previously reported miRNAs as mutually upregulated, mutually downregulated, and mutually dysregulated but not mutually upregulated or mutually downregulated, as illustrated in Figure 1.

**Figure 2:**
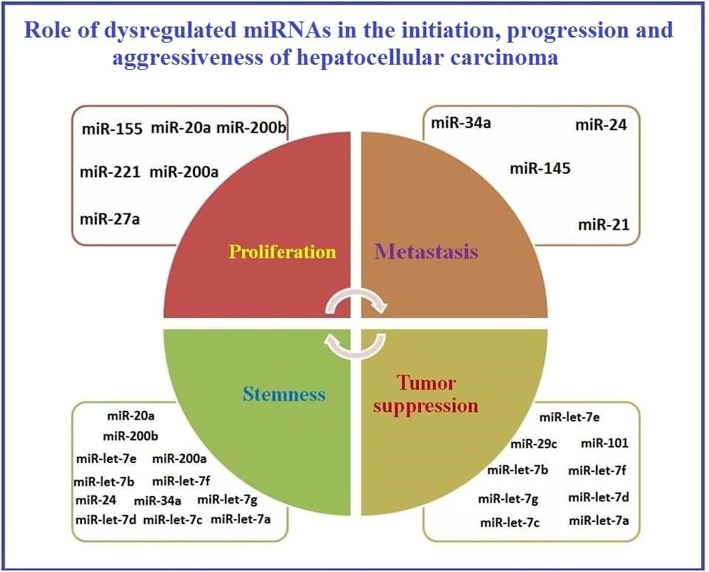
Representation of the role of dysregulated miRNAs in initiation, progression, and aggressiveness of Hepatocellular carcinoma.

A schematic presentation of the role of dysregulated miRNAs in HCC initiation, progression, and aggressiveness is presented in Figure 2.

As reported previously, MSCs aid in cancer development by enhancing the metastatic capability of tumor cells (Hill et al., 2017). This has been also reasoned by the fact that MSCs can home to tumor microenvironment mainly due to the action of stromal cell-derived factor 1 (SDF-1) (Gao et al., 2009). After homing, MSCs start to trans-differentiate into cancer associated fibroblasts mainly due to the action of TGF-beta1 (Ghaderi and Abtahi, 2018). Afterwards, cancer associated fibroblasts (CAFs) start to induce metastasis in neighboring tumor cells by inducing EMT (Wang et al., 2018). Some of the significant genes involved in such pro-metastatic signature of the tumor MSCs have been identified in lung cancer and they include GREM1, LOXL2, ADAMTS12, and ITGA11 (Fregni et al., 2018). Also, as investigated by our research group, soluble factors secreted from cancer cells when cocultured with MSCs have shown to induce cancer stem cell-like characteristics in the cocultured MSCs (El-Badawy et al., 2017). Relating the previous information, we are trying to highlight the mutual dysregulated miRNA in HCC, CSCs, and MSCs to investigate whether miRNAs play a vital role in the acquirement of MSCs to pro-metastatic characteristics or development into Cancer stem cells- like cells or even CAFs. So, in Table 2, we summarize the roles of miRNAs that are mutually dysregulated in HCC, CSC, and in MSCs. The functions of these miRNAs may provide insight into their regulatory roles in the development of cancer (Schraivogel et al., 2011). Based on these proposed functions (Table 2), we classified these miRNAs according to their roles in MSC differentiation.

**Table 2:** The roles of the mutually dysregulated miRNAs in HCC and CSC, in MSC differentiation.

**miRNA**	**Role in MSCs**
miR-let-7 family	Inhibits adipogenesis and migration of cells (Sung et al., 2013)
miR-16	Enhances myogenesis and G1 arrest (Liu et al., 2012; Wang et al., 2012)
miR-17	Enhances osteogenesis (Liu Y. et al., 2011)
miR-20a	Enhances osteogenesis (Zhang et al., 2011)
miR-21	Enhances both osteogenesis and adipogenesis but inhibits proliferation and aids in survival under hypoxic conditions (Nie et al., 2011; Kim et al., 2012; Mei et al., 2013)
miR-24	Enhances adipogenesis and inhibits osteogenesis (Sun et al., 2009; Hassan et al., 2010)
miR-27a	Inhibits osteogenesis (Schoolmeesters et al., 2009; Hassan et al., 2010)
miR-141	Inhibits osteogenesis (Itoh et al., 2009)
miR-145	Inhibits chondrogenesis (Tong et al., 2011; Martinez-Sanchez et al., 2012)
miR-155	Inhibits adipogenesis and immune regulation (Skårn et al., 2012; Xu C. et al., 2013)
miR-194	Inhibits chondrogenesis (Xu et al., 2012)
miR-200a	Inhibits osteogenesis (Thurnherr et al., 2016)
miR-221	Inhibits adipogenesis (El-Halawany et al., 2015)
miR-222	Inhibits adipogenesis (El-Halawany et al., 2015)

**Figure 3:**
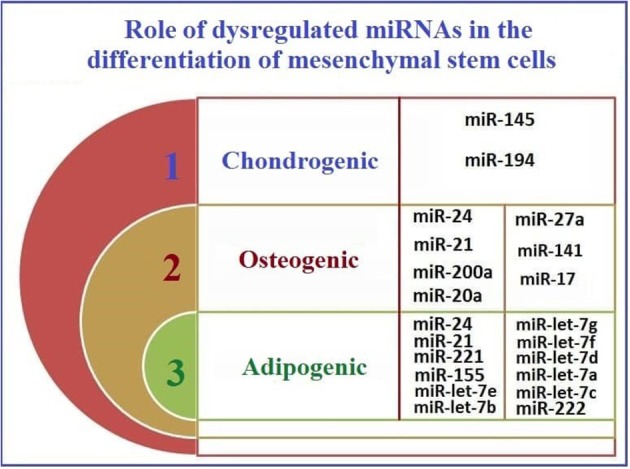
The roles of mutually dysregulated miRNAs in HCC and CSC, in MSC differentiation.

Figure 3 shows the potential pathways that may be involved in the reprogramming of MSCs and their acquisition of CSC-like characteristics after co-culture with cancer cells.

The relationship between the expression of miRNAs expression, their target genes' expression, and the fate of HCC is detailed in Table 3.

**Table 3:** The relationship among miRNAs' expression, target genes' expression, and the fate of HCC.

**miRNA**	**Expression**	**Target genes**	**Expression of target genes**	**Fate of HCC**
miR-Let-7a				
miR-Let-7b				
miR-Let-7c				
miR-Let-7d		RAS oncogenes		Suppression
miR-Let-7e				
miR-Let-7f				
miR-Let-7g				
miR-200 a		Foxa2		Enhanced proliferation and carcinogenesis
miR-200 b	Downregulation	*BMI1*	Upregulation	
miR-145		ROCK 1		Increased tumorigenesis and invasion
miR-34a		HDAC1		Inhibition of invasion and migration
miR-141		TIAM1		Inhibition of proliferation, invasion, and migration
miR-7		CDR1		Suppression
miR-424		C-myb		Inhibition of invasion and proliferation
miR-9		PPAR alpha		Increase growth and metastasis
miR-16		Bcl-2		Inhibition of proliferation, invasion, and metastasis
miR-17		TSP 1		Increased tumorigenesis
miR-18		SMAD2		Inhibition of migration
miR-20a		MCl-1		Increased proliferation and recurrence
miR-21		KLFS		Increased progression and metastasis
miR-24		P53		Increased Metastasis and invasion
miR-27a		PPAR-y		Increased proliferation
miR-495		IGFlR		Inhibition of proliferation and invasion
miR-191	Upregulation	TIMP3	Downregulation	Increased proliferation and tumorigenesis
miR-155		Oncogenesis and casein kinase 1-α (CK1-α)		Increased proliferation and tumorigenesis and decreased apoptosis
miR-221		NFkB and downstream genes such as (bcl-2/MMP-9 and VEGF)		Increased proliferation and tumorigenesis
miR-22		3UTR of CD147		–
miR-210		SMAD4-STAT6		Increased angiogenesis
miR-1246	Downregulated	CADM1	Upregulated	Increased migration
miR-29c	Downregulated	SIRT 1	Upregulated	Suppression
miR-214	Downregulated	CTNNB 1	Upregulated	Suppression
miR-215	Downregulated		Upregulated	Suppression
miR-142	Downregulated	THBS4–TGF-β	Upregulated	Increased migration, invasion, and metastasis
miR-150	Downregulated	GAB1	Upregulated	Suppression
miR-93	Upregulated	PDCD 4	Downregulated	Increased migration and invasion
miR-183	Upregulated	ETS2 and EGR1	Downregulated	Carcinogenesis
miR-185	Downregulated	DNTM1	Upregulated	Increased proliferation
miR-194	Downregulated	MAP4K4	Upregulated	Increased proliferation
miR-101	Downregulated	3′UTR of WT-PTEN	Upregulated	Promotion of apoptosis and suppression

## Dysregulated miRNA Methylation in HCC

Genome-wide abnormal DNA methylation of miRNA host genes in HCC was recently reported. One study analyzed tumor and neighboring normal non-tumor tissues in 62 HCC patients. This analysis was performed using Infinium Human Methylation Analysis Bead Chips. One hundred ten miRNAs from 64 different host genes were covered in this analysis through assessing the methylation of 254 CpG sites. Methylation levels were found to be significantly different at 54 CpG sites from 27 host genes between tumor and neighboring normal non-tumor tissues (Shen et al., 2012). In addition, the expression of three identified miRNAs were measured. MiR-10a was downregulated in tumor tissues and therefore its action on its oncogenic target genes as a tumor suppressor miRNA diminished. This decline appeared to be related to hypermethylation of the host genes. Accordingly, aberrant methylation and expression of miRNAs were considered valuable molecular biomarkers for HCC early diagnosis (Shen et al., 2012). In another study, miRNA genes, from HCC cells and normal liver hepatocytes, showed significantly different profiles of global DNA methylation. In the same study, in HCC cells, miRNAs CpG-poor regions were more commonly hypomethylated rather than being hypermethylation (He et al., 2015). Investigations using miRNA expression microarray data identified 10 dysregulated miRNAs in HCC that are regulated by DNA methylation. Of the 10 studied miRNAs, miR-23a/27a and miR-25/93/106b constituted two miRNA clusters in which five miRNAs were upregulated. On the other hand, the other five miRNAs including miR-375, miR-195, miR-497, miR-378, and miR-148a were downregulated (He et al., 2015). The cluster containing miR-25/93/106b, with upregulated expression, was required for cell proliferation including the anchorage-independent growth. It was also shown to target the E2F1 transcription factor in HCC, which inhibits apoptosis (Li Y. et al., 2009). Additionally, miR-331-3p was shown to target PHLPP, a protein that plays a central role in inducing apoptosis and reducing metastasis (Ma et al., 2010; Liu J. et al., 2011; Chang et al., 2014; Peuget et al., 2014). These data provide further evidence for the potential role of miR-331-3p in HCC metastasis. The miR-23a/27a cluster, with upregulated expression, enhanced anti-apoptotic pathways in addition to promoting cells proliferation in HCC (Huang et al., 2008). In another study, miR-429 functioned by manipulating liver tumor-initiating cells to target the RBBP4/E2F1/OCT4 axis and was upregulated in HCC due to four aberrant hypomethylated upstream sites (Li L. et al., 2015).

AEG-1 and ATG7 were found to be targets for miR-375, one of the previously mentioned epigenetically downregulated miRNAs, which makes miR-375 a tumor suppressor miRNA in HCC (Chang et al., 2012; He et al., 2012). When overexpressed, miR-375 inhibited both migration and invasion in HCC (He et al., 2012). Cell growth was also inhibited by the action of the miR-195/497, which targeted vital cell cycle regulators, leading to G1 arrest in HCC (Furuta et al., 2013). As reported using a bioinformatics approach, CDK4, which is involved in chemotherapy-mediated tumor cell apoptosis, was predicted to be a potential miR-195 target (Yang et al., 2011). MiR-378 suppressed HCC tumor growth, which was originally caused by HBV infection. MiR-378 was found to directly target the insulin-like growth factor 1 receptor (IGF1R) (Li et al., 2013). IGF2BP1, highly involved in liver cancer progression by promoting metastasis, was reported as an expected miR-378 target (Gutschner et al., 2014). Acting as a tumor suppressor miRNA by targeting c-Met which is an oncogene, miR-148 has also been shown to target DNMT1 in hepatocytes leading to the induction of liver-specific phenotype (Gailhouste et al., 2013). MiR-148a is among the five epigenetically downregulated miRNA by methylation and since DNMT1 and DNMT3B are considered to be two of its targets and responsible for its methylation, miR-148a together with DNMT1 and DNMT3B constitute a positive feedback mechanism which in the case of HCC leads to miR-148a downregulation (Duursma et al., 2008; Pan et al., 2010).

Due to several reports of miRNA hypomethylation in HCC, DNA hypomethylation was shown to play a significant role in miRNA regulation. Moreover, DNA methylation might be responsible for the abnormal expression of these miRNAs. Accordingly, further studies on these dysregulated miRNAs are needed (He et al., 2015).

In HCC, miRNAs have been shown to be regulated through epigenetic markers, specifically by DNA methylation. In Table 4, we review reported methylation-regulated miRNAs in HCC. One of the few studies in this area reported that methylation of miR-203 in CSCs plays a role in EMT and increases cancer progression (Taube et al., 2013). A summary of the available data concerning the epigenetic control of miRNA expression in HCC via methylation is provided in Table 4. Also, Table 4 shows the role the methylation of these miRNAs plays in the progression of HCC.

**Table 4:** miRNAs regulated by methylation in HCC.

**miRNA**	**Function/target**	**Impact of methylation**	**Status**	**Expression upon methylation**
miR-148a	Acts as a tumor suppressor (Pan et al., 2014)	Enhanced tumorigenesis and HCC progression	Hypermethylation (He et al., 2015)	Downregulation
miR-375	Acts as a tumor suppressor by inhibiting metastasis (Xie D. et al., 2017) 34	Increased metastasis and HCC progression	Hypermethylation (He et al., 2015)	Downregulation
miR-195	It acts as a tumor suppressor through metastasis inhibition (Wang M. et al., 2015)	Enhanced tumorigenesis and HCC progression	Hypermethylation (He et al., 2015)	Downregulation
miR-497	It acts as a tumor suppressor by inhibiting metastasis angiogenesis (Yan et al., 2015)	Enhanced angiogenesis and metastasis	Hypermethylation (He et al., 2015)	Downregulation
miR-378	Acts as a tumor suppressor (Li et al., 2013)	Enhanced proliferation	Hypermethylation (He et al., 2015)	Downregulation
miR-106b	Targets DAB2 (Sun et al., 2018)	Proliferation and migration	Hypomethylation (He et al., 2015)	Upregulation
miR-25	Inhibits RhoGDI1 (Wang C. et al., 2015)	Promotion of both migration and invasion	Hypomethylation (He et al., 2015)	Upregulation
miR-93	Targets PDCD4 (Ji et al., 2017)	Enhanced metastasis and invasion	Hypomethylation (He et al., 2015)	Upregulation
miR-23a	Acts as an oncomiR (Bao et al., 2014)	Onset of HCC	Hypomethylation (He et al., 2015)	Upregulation
miR-27a	Targets the peroxisome proliferator-activated receptor γ gene (Li S. et al., 2015)	Increased proliferation capacity	Hypomethylation (He et al., 2015)	Upregulation
miR-10a	Is reported to have several functions. It can promote migration and invasion, while inhibiting angiogenesis capacity in HCC and reduce metastasis capability by targeting Â1-integrin and MMP-2 (Tang, 2013)	After hypermethylation, metastasis and angiogenesis are expected to be reduced. On the other hand, invasion, and migration capabilities would be reduced	Hypermethylation (Shen et al., 2012)	Downregulation
miR-10b	Acts by targeting CSMD1, RhoC, uPAR, and MMPs (Liao et al., 2014; Zhu et al., 2016)	Hypermethylation of this miRNA is expected to reduce levels of migration, proliferation, and invasion potential	Hypermethylation (Shen et al., 2012)	Downregulation
miR-196b	Targets FOXP2 (Yu et al., 2018)	Decreased metastasis, proliferation, and migration potential	Hypermethylation (Shen et al., 2012)	Downregulation
miR-1	It acts as an oncomiR (Hu et al., 2015)	Decreased proliferation and migration potential	Hypermethylation (Datta et al., 2008)	Silenced
miR-124	Acts as a tumor suppressor by targeting Baculoviral IAP repeat containing 3 (BIRC3) gene (Cao et al., 2018)	Promotion of proliferation and migration potential	Hypermethylation (Furuta et al., 2010)	Silenced
miR-125b	Acts as a tumor suppressor by targeting (TAZ) transcriptional co-activator (Li J. et al., 2015)	Increased migration and invasion	Hypermethylation (Alpini et al., 2011)	Silenced
miR-203	Acts as a tumor suppressor by targeting survivin (Wei W. et al., 2013)	Increased proliferation potential	Hypermethylation (Furuta et al., 2010)	Silenced
miR-1247	Acts as a tumor suppressor with Wnt3 being its target (Chu et al., 2017)	Increased proliferation and invasion potential	Hypermethylation (Anwar et al., 2013)	Downregulation
miR-132	Acts as a tumor suppressor by targeting PIK3R3 (Hu et al., 2015)	Increased proliferation, invasion, and migration potential	Hypermethylation (Wei X. et al., 2013)	Downregulation
miR-320	Acts as a tumor suppressor where c-Myc is its target (Xie F. et al., 2017)	Increased proliferation and invasion	Hypermethylation (Shen et al., 2012)	Downregulation
miR-596	No data available	No data available	Hypermethylation (Anwar et al., 2013)	Downregulation
miR-663	Inhibits proliferation by targeting the HMGA2 gene (Huang et al., 2016)	Increased proliferation and invasion	Hypermethylation (Potapova et al., 2011)	Downregulation
miR-9	Different data are reported: It may act as a tumor suppressor role by targeting TAZ (WWTR1) (Higashi et al., 2015). Moreover, it targets KLF17 and increases the migration and invasion properties (Sun et al., 2013)	Data reported that hypermethylation could lead to HCC progression (Higashi et al., 2015), while other reports suggested that hypermethylation can lead to reduced migration and invasion properties (Sun et al., 2013)	Hypermethylation (Anwar et al., 2013)	Downregulation

Data in Table 4 shows some consistent pattern between the role of different miRNAs and their methylation patterns. To illustrate, miRNAs that act as tumor suppressors are hypermethylated while oncomiR are hypomethylated, which finally leads to HCC progression. On the other hand, in HCC, some miRNAs showed an opposite pattern. MiR-10a, miR-10b, miR-9, and miR-196b have been shown to be hypermethylated and their hypermethylation state would lead to reduced tumorigenesis. Therefore, further studies are needed to investigate other roles for these specific miRNAs, and especially the function of miR-596 in HCC progression.

## Conclusion

Several studies showed how tumor microenvironment enhances cancer development and progression (Whiteside, 2008; Wang et al., 2017; Klymenko and Nephew, 2018). Studies from our laboratory have shown that soluble HCC factors play a vital role in the induction of chemoresistance properties in human bone marrow (hBM)-MSCs and trigger their transformation into CSC-like cells (El-Badawy et al., 2017). However, the mechanism of this transformation remains unclear. Although the initiation of HCC is known to be preceded by cirrhosis, the initiation mechanism itself is thought to involve CSCs. CSCs were proposed to be responsible for chemoresistance and relapse in most cancers. Previous data reported by our research group highlighted the role of CSCs in HCC initiation and progression (El-Badawy et al., 2017). Studies also demonstrated that liver CSCs are associated with liver cancer metastasis and relapse and that CSCs play a substantial role in the resistance of liver cancer to conventional treatment (Xu et al., 2009). These data indicate that targeting CSCs as a potential therapeutic approach for liver cancer holds huge promise for improving the treatment outcomes (Lou et al., 2018). Since reprogramming events responsible for the transformation and initiation processes are mainly controlled by epigenetic modifications, determining the role of such epigenetic fingerprints, including miRNAs and their methylation, in initiation, relapse, and chemoresistance in HCC and CSCs is central to understanding tumor biology and developing effective therapies.

Herein, we are presenting growing evidence supporting the central role of miRNAs in many biological processes (Brennecke et al., 2003; Ambros, 2004). In addition, miRNA dysfunction causes the development of diverse cancers (Iorio and Croce, 2009; Negrini et al., 2009). Recent studies show that HCC is associated with altered levels of miRNAs (Murakami et al., 2006; Jiang et al., 2008; Wong et al., 2008). Moreover, several miRNAs, such as miR-195, miR-122, miR-101, and miR-121, have been reported to regulate cell invasion, migration, apoptosis and growth (Fornari et al., 2008; Wang et al., 2008; Su et al., 2009). These findings suggest that dysregulation of miRNA may be attributed to hepatocarcinogenesis (Xiong et al., 2010).

Accumulating data on the expression profiles, roles, and regulation of miRNAs are essential for designing effective stem cell therapies for HCC. In this review, we highlighted the dysregulated expression of relevant miRNAs in HCC and CSCs. Based on the classification of miRNAs as mutually upregulated, mutually downregulated, or mutually dysregulated, we proposed their roles in cancer progression. Despite the lack of data on miRNA expression in MSCs, four miRNAs have dysregulated expression in HCC, CSCs, and MSCs. Based on their functions in MSCs, these miRNAs have been shown to mainly affect differentiation. It is clear, however, that more research is required on the expression profiles of miRNAs in MSCs under physiological conditions and from different tissue sources. Such studies are essential for determining how MSCs regulate and interact with cancer cells and CSCs in HCC.

In the context of clinical applications, miRNAs could represent an opportunity to develop safe strategies for achieving early diagnosis, monitoring disease status, and improving the effectiveness of non-invasive HCC treatment (Valeri et al., 2009). Several studies have shown the effect of miRNAs on enhancing the sensitivity of liver CSCs to treatment. Many dysregulated miRNAs in liver CSCs exert their roles by binding to specific target genes that are key molecules in many pathways. Targeting these miRNAs, their targeted genes, or respective pathways may thus effectively target CSCs, and disturb their role in metastasis, recurrence, and resistance to therapy (Lou et al., 2018). Together with conventional treatment, targeting specific miRNAs involved in tumor progression in combination therapy represent an attractive approach to multifactorial effective therapy to liver cancer (Tao et al., 2018). Although no miRNAs-based drugs are available in current clinical practice (Lou et al., 2018), the antitumor efficiency of modern anticancer drugs like sorafenib on HCC was significantly increased *in vivo* upon delivery of miR-122-exosome to the tumor (Blechacz and Gores, 2008; Lou et al., 2015). Such enhancement is promising to patients of unresectable HCC whose treatment with sorafenib was of limited efficacy, by prolonging survival for only 3 months (Blechacz and Gores, 2008; Kane et al., 2009). Investigating the expression signature of those candidate miRNAs in HCC diagnosis, prognosis, metastasis, and recurrence and determining how these miRNAs genetically and epigenetically regulate the transformation of somatic stem cells to a more chemoresistant phenotype is needed for translation to clinical practice (Valeri et al., 2009; El-Badawy et al., 2017).

## Author Contributions

Each author has substantially contributed to conducting this study and drafting this manuscript. MN and RS wrote the manuscript. MN, ME, and SE analyzed data and moderate figures and tables. While, NE-B contributed in writing and editing of the manuscript.

### Conflict of Interest

The authors declare that the research was conducted in the absence of any commercial or financial relationships that could be construed as a potential conflict of interest.
